# Dynamics of daily vertical migration in mesopelagic fish communities across the Southern Ocean

**DOI:** 10.1098/rspb.2025.2264

**Published:** 2026-03-04

**Authors:** Zhengxin (Jasmine) Yang, Sophie Fielding, Jennifer J. Freer, Geraint A. Tarling, Ryan Alexander Saunders, Martin J. Genner

**Affiliations:** ^1^ School of Biological Sciences, University of Bristol, Bristol, UK; ^2^ British Antarctic Survey, Cambridge, Cambridgeshire, UK

**Keywords:** abundance, biomass, diel vertical migration, Myctophidae, lanternfishes, Antarctica

## Abstract

Mesopelagic fishes transport a vast biomass of organic material between surface and deep waters through diel vertical migration (DVM), which plays a crucial role in both food web functioning and deep-ocean carbon sequestration. However, there is considerable uncertainty about which species perform DVM and the extent of their migrations. This uncertainty is particularly pronounced in polar regions, where inferences of migratory behaviour have typically been made using spatially and temporally constrained data. Here, we use a recently compiled multi-decadal net trawl database to conduct a circumpolar-scale analysis of DVM behaviour in lanternfishes (Myctophidae), the dominant mesopelagic fishes of the Southern Ocean. Generalized additive models show that all eight of the most abundant myctophid species perform vertical migrations, but the species differ in the distance of migration. Only a proportion of each population undergoes DVM at a given time, demonstrating variable DVM behaviour among individuals. We estimate from our models that of a total biomass of 48.3 million tonnes of lanternfishes across the Southern Ocean, 14.2 million tonnes undergo daily vertical migrations. This study reveals the DVM pattern of key myctophid species in previously unattainable detail and provides a framework to better understand their critical role in the Southern Ocean ecosystem.

## 1. Introduction

Mesopelagic fish, typically residing at oceanic depths between 200 m and 1000 m, are the most abundant vertebrate group [1]. In the Southern Ocean, defined here as the ocean south of 40° S, the mesopelagic fish community is dominated by lanternfishes (Myctophidae) in terms of species richness, abundance and biomass [2–4]. They occupy a central role in the food web by linking secondary producers and higher predators [5,6], feeding on a range of amphipods, copepods and euphausiids—including Antarctic krill [6–10]. In turn, they are consumed by squid, flighted seabirds, penguins, mammals and predatory fishes [4], supporting the large numbers of top predators of the Southern Ocean [5,11,12].

A key characteristic of myctophids is their diel vertical migration (DVM)—the daily migration between surface waters at night for feeding and deeper waters in the day to avoid visual predators. Myctophid DVM has the potential to contribute significantly to carbon export by actively transporting carbon ingested at the surface to the deep, where it may be sequestered after being released through respiration, excretion or carcass decomposition (active flux; reviewed in [13]). Yet only two studies to date have attempted to quantify active carbon export by myctophids in the Southern Ocean, estimating a fish-based export equivalent to 27–143% of the passive carbon flux (gravitational sinking of organic matter from surface water to the deep) [14,15], making this a key area of uncertainty in biogeochemical modelling [16]. A major challenge in quantifying carbon export is obtaining accurate estimates of myctophid abundance [14]. This is complicated by the need to account for variation in abundance with both time of day and ocean depth due to DVM behaviour [13]. Understanding the DVM of myctophids is also crucial for resolving the Southern Ocean trophodynamics, as their vertical distribution pattern governs prey availability for surface-dwelling predators [17].

Southern Ocean myctophids are particularly difficult to study due to the inaccessibility of polar pelagic habitats, resulting in most studies of their vertical distributions using regionally specific data that are limited in spatial and temporal scales [2–4,10,18–20]. This is potentially problematic as DVM behaviour may vary spatially and temporally within and among species [2,19,21–23]. Moreover, studies investigating myctophid ecology tend to be based on data collected by a single type of sampling gear, so they are potentially biased in the size of fishes analysed due to differing catch efficiencies among gears [24–26]. A broader macro-scale perspective of Southern Ocean lanternfish DVM that encompasses multiple gear types, therefore, has considerable potential to provide key details on the dynamics of vertical migration behaviour of this species group and to provide valuable insight into the relevance of lanternfish DVM for ecosystem-scale ecological processes.

In this study, the DVM behaviour of eight ecologically important myctophid species in the Southern Ocean was analysed using net-based survey data obtained from the taxonomically specific database Myctobase [27]. With large spatial and temporal coverage, this dataset has previously been used to estimate circumpolar abundances of myctophids with species distribution models, which have indicated the presence of DVM behaviour in some species [28]. Here, the DVM behaviour of key species was examined more specifically using generalized additive models (GAMs) to estimate how the abundance of each species changes in relation to time of day and depth. Our analyses provide the first quantitative biomass estimates of both species-specific and community-wide myctophid DVM at the scale of the Southern Ocean, and the results are discussed with reference to both oceanic trophodynamics and carbon sequestration.

## 2. Methods

### (a) Myctobase dataset

Abundance records of myctophid fish from 4652 trawls were downloaded from Myctobase—a compilation of Southern Ocean pelagic net trawl data collected by seven national research institutes [27]. The dataset contains records of research cruises from 1991 to 2019, with most surveys conducted in the summer months (electronic supplementary material, figure S1). Sampling extended from 48° to 70° S and focused on the Indian and Atlantic sectors of the Southern Ocean (figure 1). Trawls were conducted from the surface to a depth of 2000 m, with most sampling events taking place in the upper 200 m of the water column (electronic supplementary material, figure S1). Opening–closing net systems were used for data collection in most cases to sample discrete depth ranges. The exception was the International Young Gadoid Pelagic Trawl (IYGPT) trawls that lacked a midwater opening/closing (MIDOC) device, which may have caught some fishes during the ascent and descent of the net from non-target depth, though such catches should be minimal [31]. Across the Myctobase dataset, sampling methodology and equipment differed between cruises, research teams and scientific institutes (electronic supplementary material, table S1). Woods *et al.* [27] provide detailed descriptions of the Myctobase data structure and survey sampling methods. The IYGPT nets used in Myctobase varied in dimensions across the scientific institutes and will be referred to with their mouth-opening size henceforth.

**Figure 1.**
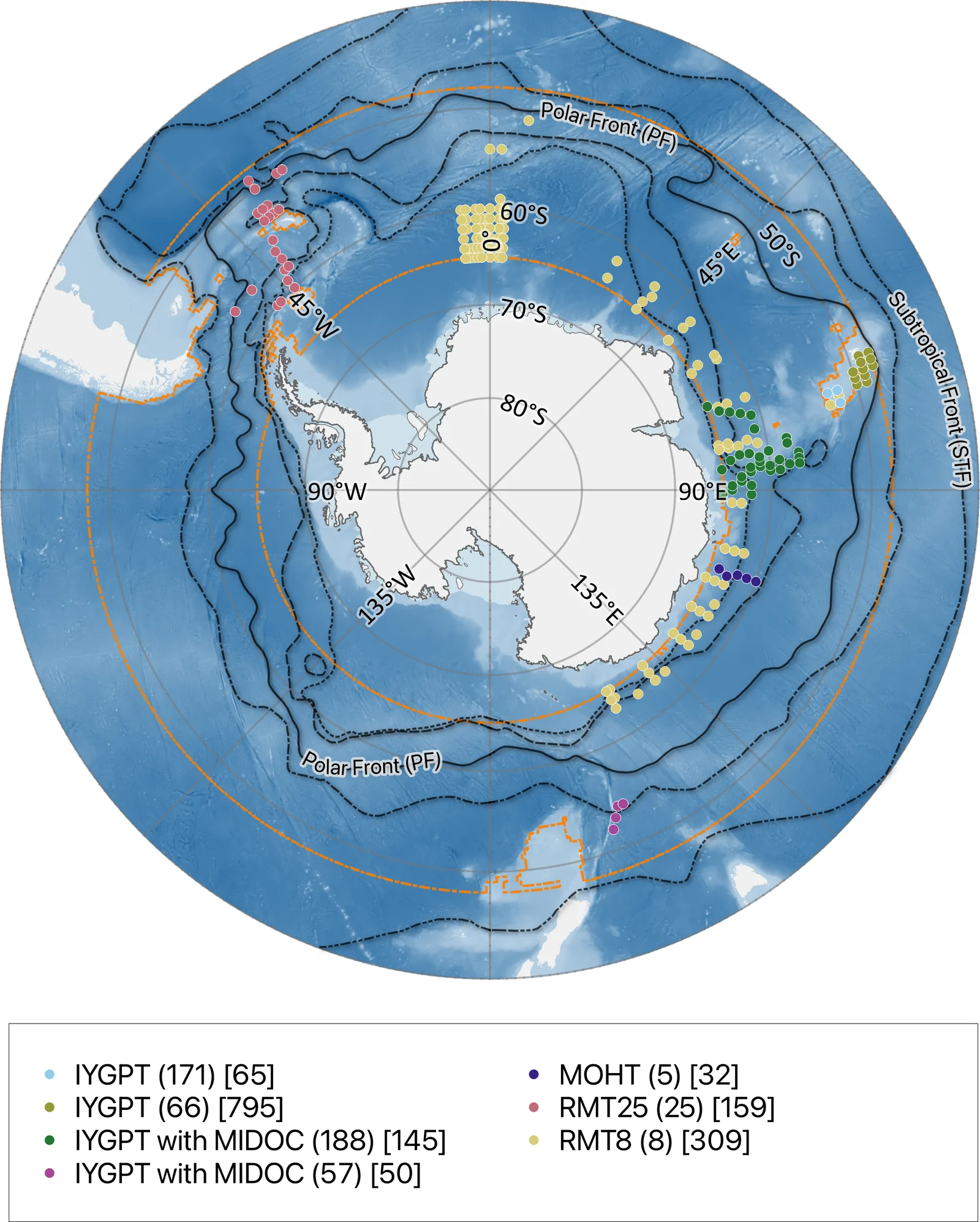
The sampling location of the pelagic net trawls data used in this study taken from Myctobase [27], post data filtration. Points are colour-coded by the types of net used: International Young Gadoid Pelagic Trawl (IYGPT); midwater opening/closing cod-end device (MIDOC); Matsuda-Oozeki-Hu trawl (MOHT); rectangular midwater trawl (RMT). In the key, the values in parentheses represent the size of net mouth opening in m^2^, while the values in square brackets indicate the number of trawls taken by each net type. The orange lines delimit the area used to calculate total myctophid abundance. Climatological ocean fronts, as defined by Orsi *et al.* [29], are shown by the black lines, starting from the innermost line: southern boundary of the ACC (SBdy); southern Antarctic Circumpolar Current Front (sACCF); Polar Front (PF); Subantarctic Front (SAF); Subtropical Front (STF). Figure produced using the Quantarctica 3.2 project [30].

The following data from each trawl were extracted: the number of fishes caught in each trawl per species, time of sampling, latitude and longitude of trawl locations, maximum and minimum depth sampled, volumes of water filtered in trawls and measurements of the sampling net used. The standard length and wet weight of individual fish were also extracted, although these data were not available for every trawl.

Eight of the most abundant and ecologically important myctophid species in the Southern Ocean were the focus of this research: *Electrona antarctica*, *Krefftichthys anderssoni*, *Gymnoscopelus braueri*, *Protomyctophum bolini*, *Gymnoscopelus nicholsi*, *Gymnoscopelus fraseri*, *Protomyctophum tenisoni* and *Electrona carlsbergi* (electronic supplementary material, table S2). These eight species collectively comprise >95% of the total myctophid catch in Myctobase. With the exception of *E. antarctica* and *E. carlsbergi*, all species exhibited the highest abundance north of the Polar Front within the Subantarctic zone (electronic supplementary material, figures S2 and S3). *E. antarctica* was the most frequently encountered species across all trawls (electronic supplementary material, table S2) and was present throughout the Southern Ocean. In some species, the standard length of the fish varied along latitude and sampling depth (results and discussions in electronic supplementary material, figures S4 and S5).

### (b) Data quality control

The dataset was first filtered to remove trawls with missing information on sampled depth and filtered water volume. Where possible, missing information was filled in according to the original methodology paper of each dataset. Data collected by target trawls (trawling at locations with acoustically detected fish aggregations) were also excluded. Where a species was absent from a trawl, it was assigned an abundance of 0 for that particular trawl (zero-count). Sampling depth was calculated as the mean of the maximum and minimum sampling depth. Trawls with depth intervals exceeding 300 m were excluded. The exception to this was samples collected using the 66 m^2^ mouth-opening IYGPT net (795 trawls), which only had values for maximum sampling depth. This net used horizontal sampling, where the net was towed at a constant depth, so the maximum sampling depth was used directly as the sampling depth. For information on inferring sampling depths using the IYGPT net, see [31].

Standardized abundance estimates depend, in part, on accurate reporting of the volume of water filtered during each trawl. The reported volumes of water filtered were checked for outliers by fitting a linear model of volume filtered against haul duration for each cruise, separated by the types of net used. An outlier was defined as a reported value of volume filtered exceeding its predicted value by three times the residual standard deviation; those outlier values were converted to the predicted value from the linear model (electronic supplementary material, figure S6). For cruises without haul duration data, any reported volumes exceeding three standard deviations from the mean were classed as outliers and converted to the mean volume for that cruise. A total of 15 trawls were corrected for volume values.

### (c) Data filtration

For each species, trawls were removed for DVM analysis if they were outside the 90th percentile of the species’ latitudinal range of all occurrences (electronic supplementary material, figure S2), which enabled us to only focus on locations where the species was most abundant. Data collected using Isaacs–Kidd Midwater Trawl nets (2105 trawls) were removed from the DVM analysis, as this net was only used to sample surface waters (<200 m), so it was not suitable for evaluating changes in myctophid abundance with depth. Similarly, trawls south of 65° S, which also only sampled surface waters, were excluded from analyses. This mostly affected *E. antarctica,* as other species were largely absent south of 65° S (electronic supplementary material, figure S2). Following these data filtration steps, a total of 1555 trawls were used in the DVM analyses (figure 1).

### (d) Modelling diel vertical migration behaviour

The DVM behaviour of myctophids was modelled using GAMs. All analyses were conducted in R v. 4.3.1 [32] with GAMs built using the package ‘mgcv’ v. 1.8-42 [33] employing restricted maximum likelihood estimation. To identify a consistent model structure across species, models were generated that predicted either the untransformed fish abundance or the log*_e_*-transformed fish abundance for each species, using either Tweedie (tw) or negative binomial (nb) distributions to account for zero-inflated data. Fish abundance was calculated as the number of individuals per 1000 m^3^ of filtered water for each trawl. The fits of these models were compared using AIC, *R*^2^ and likelihood statistics. Models fitted to log*_e_*-transformed abundance using Tweedie data distribution were most optimal (electronic supplementary material, figure S7 and table S3), using the following structure:


$$\begin{array}{r} log_{e}Abundance∼ti(time,\;depth,\;k=c(5,\;5),bs=c(cc,\;tp)\\ +s(time,\;k=5,bs=cc))+s(depth,k=5)+net\;effect \end{array}$$

The main effects ($s\left(time\right)$ and $s\left(depth\right)$) and the interaction term ($ti\left(time,depth\right)$) were modelled independently to allow the identification of species that do not undergo DVM (i.e. no interaction between time and depth). Penalized thin plate regression splines were used as the basis unless specified (‘cc’ = cyclic basis). The time at sampling was classified into four diel groups based on the solar position (angle of the sun in relation to the horizon) as defined in [27]: dawn (−12° to 12° before midday), day (≥13°), dusk (12° to −12° after midday) and night (≤−13°). Solar position was used to account for latitudinal and seasonal differences in day length. Diel groupings were used to ensure adequate sample sizes across depth-time combinations since there was limited temporal coverage of deeper water samples (electronic supplementary material, figure S1). The diel group was then converted to a continuous variable (to 1, 2, 3 and 4, respectively) to allow the use of a cyclic basis function for modelling the time of the day. The *k*-value (number of basis functions, or ‘knots’) for time was chosen to reflect the four time groups, and boundary knots were placed at 0.5, 1.5, 2.5, 3.5 and 4.5 to ensure the distance between dawn (1) and night (4) was the same as the distance between other times. A specific *k*-value of 5 was used for depth to keep all models consistent between species. This value was found to reflect the variation in data without overfitting in preliminary tests.

A parametric variable of neteffect was included in the model to standardize abundance across the different sampling methods used to collect the fish. This variable consisted of the first principal component (PC1) calculated from the normalized values of net mouth opening size, codend mesh size and mean tow speed (electronic supplementary material, table S1), all of which are known to affect catch efficiency [24,25]. A principal component was used as all three variables were significantly correlated with each other (Spearman’s correlation, *p* < 0.001 for all three pairings); this PC1 explained 64% of the variance among the three variables.

Models of each species were then used to calculate the proportion of the sample population performing DVM. First, the abundance estimates between 0 and 1000 m depth were extracted from the model at a metre-scale resolution for both daytime and night-time. These abundance estimates were back-transformed from the log scale before any calculations. Net effect was set as 0 for model predictions. For each species, the time group representing daytime was set as either 1.5 or 2, depending on which value had the higher abundance estimate. Similarly, the time group representing night-time was chosen as 3.5 or 4, keeping the time difference between day and night groups equal to two units. Abundance estimates were lower in the daytime than night-time, probably due to daytime net avoidance [2,3]. Therefore, to enable consistency, daytime abundance estimates were inflated equally across all depths so that the total depth-integrated abundance was equal for day and night (electronic supplementary material, figure S8). To establish whether an organism was surfacing or diving in DVM, a threshold depth was set where organisms were identified as having surfaced when above the threshold or dived when below the threshold. This was defined as the midpoint between the median depths of daytime and night-time vertical distribution. The proportion of the population performing DVM was defined as the proportion of the total population excluding non-migratory populations (i.e. individuals remaining at surface water in daytime and individuals remaining in deep water at night-time) and was calculated with the following equation:


$$DVMproportion_{i}=\;\frac{NightAbund_{i}-\;\left(DayAbund_{i}\geq \;Z_{i}\right)-\left(NightAbund_{i}\;<\;Z_{i}\right)}{NightAbund_{i}},$$

where *DayAbund* and *NightAbund* are the integrated abundance estimates during daytime and night-time, respectively, and *Z_i_* is the calculated DVM threshold depth for species *i*.

### (e) Estimating total and migratory biomass of Southern Ocean myctophids

The overall pattern of myctophid DVM in the Southern Ocean was analysed by fitting a GAM model to all eight species (hereafter referred to as the ‘all-species GAM’). This used the above GAM equation with abundance data calculated by summing the eight study species’ abundances in each trawl. All trawls north of 65° S were included in this abundance data without cropping at the 90th percentile distribution range of each species, resulting in the use of data from all 1555 trawls. This model was then used to estimate the total abundance of myctophids in the Southern Ocean, assuming equal DVM pattern and abundance across the Southern Ocean.

First, the night-time abundance per 1000 m^3^ was predicted at each 1 m depth interval for the 1–1000 m depth range. This was then summed and divided by 1000 to calculate the depth-integrated abundance of myctophids per square metre. The night-time prediction was used to reduce the chance of net avoidance behaviour affecting the abundance estimate. Ocean area was obtained using the General Bathymetric Chart of the Oceans (GEBCO) gridded database [34] and selecting areas with ≥1000 m water depth between 48° and 65° S, to only include regions with the full depth range within the modelled region (figure 1). The total myctophid abundance for this region was calculated by multiplying the depth-integrated abundance per area by the total ocean area.

To obtain the total biomass for each species, their total abundance (${TAbund}_{i}$) was calculated from the total myctophid abundance estimate (${TAbund}_{all}$) using


$$TAbund_{i}=TAbund_{all}\times prop_{i},$$

where ${prop}_{i}$ is the proportion of species *i* in the overall myctophid community of the study species. To account for differing sample sizes across latitudes, this proportion was obtained by calculating the mean abundance of each species at a 1° latitude interval (electronic supplementary material, figure S2) and then averaging across 48–65° S. The total myctophid biomass in the Southern Ocean was then calculated by


$$TBiomass_{all}\;=\sum_{i=1}^{n}TAbund_{i}\times ww_{i},$$

where ${ww}_{i}$ is a model-estimated average wet weight of species *i* to account for variation in fish size across the sample. This was calculated by fitting species-specific linear models of fish standard length against variables known to affect fish size: latitude [23,35,36], depth [3,19,21] and net effect [24,25] (the same variable included in the individual species models mentioned above; electronic supplementary material, table S4). The standard length at the median sampled latitude and depth with net effect set as 0 was then predicted from the model for each species, based on the assumption that the average biomass from trawls at this position is representative of the species across the sampled area. This was then converted to wet weight using a standard allometric weight–length relationship equation calculated for each species: W = *a*L*b* [37] (electronic supplementary material, table S5). The confidence interval of total myctophid biomass was obtained from the confidence interval of the myctophid abundance estimates alone and does not include variance from the standard length and wet weight calculation. To estimate the total migratory biomass, the proportion of the population undertaking DVM was calculated from the all-species model output and multiplied by the total myctophid biomass.

## 3. Results

### (a) Diel vertical migration behaviour of myctophid species

The species-specific GAMs explained 13–26% of the model variance in abundance and had statistically significant interactions between times of day and ocean depth in all species (electronic supplementary material, table S6), consistent with DVM behaviour. Visualization of GAM predictions showed DVM patterns in all eight species, with deeper vertical distributions in daytime than night-time (figure 2; electronic supplementary material, figure S8). Higher variability in model prediction was shown towards the deeper water (electronic supplementary material, figure S8). Except for *P. bolini*, night-time abundance was higher than daytime abundance (table 1).

**Figure 2.**
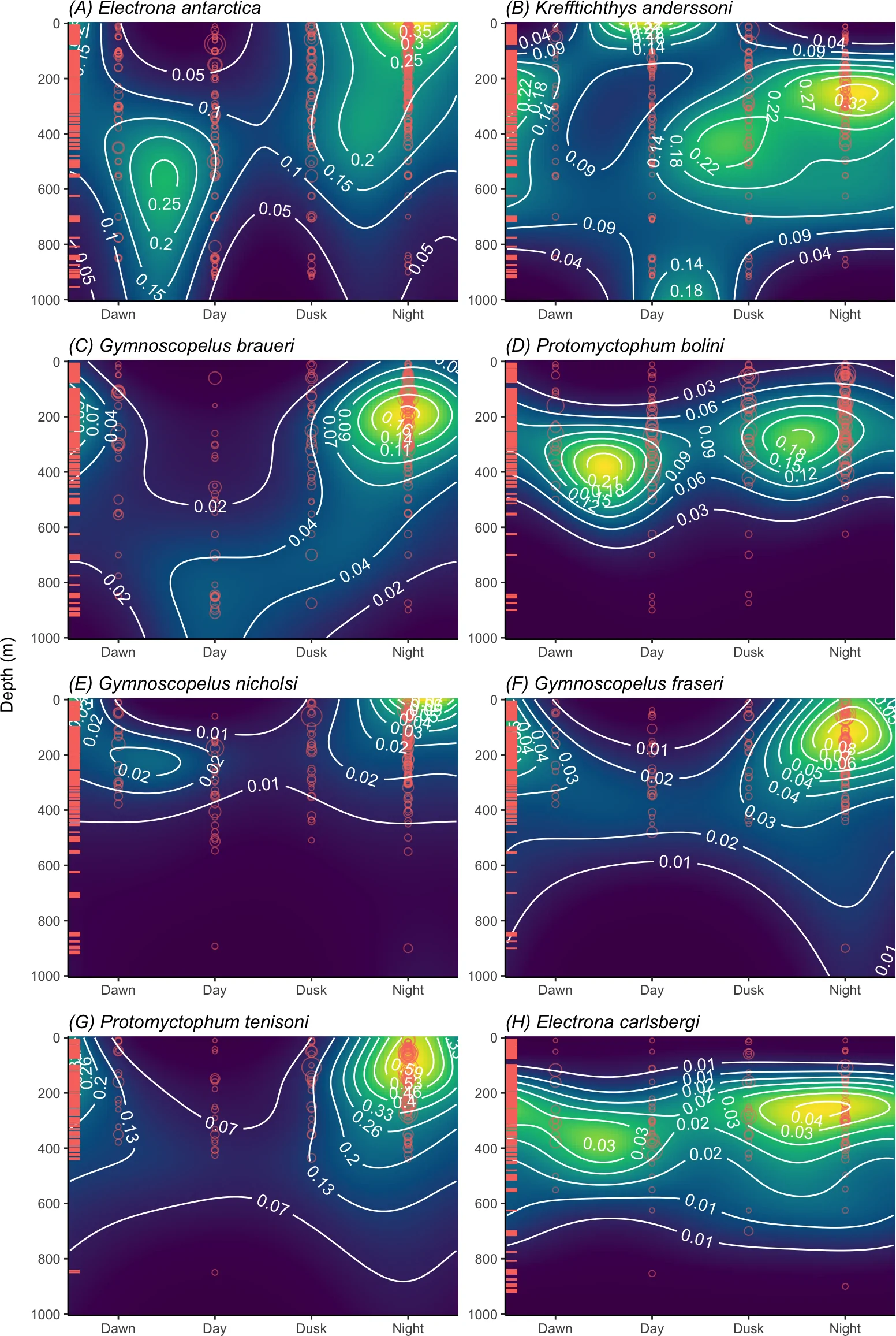
Abundance (individuals per 1000 m^3^) of myctophid species predicted using species-specific GAMs across the studied ocean depth range (*y*-axis) at different times of the day (*x*-axis). The rug on the left-hand side of each plot shows the density of data used to build the model for each species. The mean abundances calculated from raw data are shown by circles over the heatmap with size reflecting abundance.

**Table 1.** DVM patterns predicted by species-specific GAMs and all-species GAM. Central depth is the median depth of a species’s vertical distribution. Abundance values given are the depth-integrated abundances (1–1000 m) with 95% CI shown in the parenthesis (calculated as the sum of lower CI at each 1 m depth interval and the sum of upper CI at each 1 m depth interval, respectively). Threshold depth is the depth that must be passed in order to be classified as undertaking DVM: individuals located below the threshold depth in daytime were identified as descending, and individuals located above the threshold in night-time were identified as ascending. DVM proportion is the proportion of the population estimated to undergo DVM.

species	central depth (m)		abundance (ind. m^−2^)		threshold depth (m)	DVM proportion (%)
	day	night	day	night		
*Electrona antarctica*	602	372	0.159 [0.080, 0.353]	0.164 [0.110, 0.251]	487.0	34.0
*Krefftichthys anderssoni*	493	372	0.117 [0.070, 0.210]	0.144 [0.092, 0.250]	432.5	15.4
*Gymnoscopelus braueri*	739	269	0.027 [0.016, 0.045]	0.068 [0.051, 0.095]	504.0	59.2
*Protomyctophum bolini*	397	296	0.071 [0.035, 0.162]	0.064 [0.043, 0.098]	346.5	30.1
*Gymnoscopelus nicholsi*	269	167	0.005 [0.003, 0.008]	0.013 [0.009, 0.022]	218.0	24.3
*Gymnoscopelus fraseri*	383	232	0.009 [0.004, 0.020]	0.036 [0.022, 0.073]	307.5	29.9
*Protomyctophum tenisoni*	441	221	0.042 [0.018, 0.148]	0.242 [0.136, 0.552]	331.0	36.4
*Electrona carlsbergi*	388	352	0.011 [0.005, 0.023]	0.015 [0.009, 0.027]	370.0	9.1
All species^a^	576	364	0.349 [0.217, 0.607]	0.448 [0.328, 0.625]	470.0	29.3

a Predictions from an independent all-species GAM, rather than the sum of the eight species-level models.

*E. antarctica* was most abundant above 400 m depth at night and below 600 m depth in the day (figure 2; table 1). However, this species was present throughout the entire water column at all times, with 34.0% of individuals estimated to be performing DVM at any one time. *K. anderssoni* displayed unique vertical distributions, concentrated around 400 m depth at night-time, while exhibiting a more even distribution across the entire depth range during daytime, including daytime surface-caught individuals (figure 2; table 1). In total, 15.4% of *K. anderssoni* individuals were estimated to perform DVM. *G. braueri* displayed the longest migration distance with high abundance above 300 m depth at night and below 700 m depth in the day (figure 2; table 1). This species also had the highest proportion of individuals performing DVM at 59.2%. *P. bolini* was most abundant around 300 m at night and around 400 m in the day, with 30.1% of the individuals estimated to undergo DVM. Its vertical distribution range was largely confined to depths between 100 m and 600 m. *G. nicholsi* was mainly confined to epipelagic layers, distributed around 150 m depth at night and around 250 m depth in the day with 24.3% of the population undergoing DVM (figure 2; table 1). *G. fraseri* was most abundant above 250 m depth at night and around 400 m depth in the day, with 29.9% of the individuals estimated to be performing DVM (figure 2; table 1). *P. tenisoni* displayed a relatively long migration distance, concentrated around 200 m depth at night and around 450 m in the day (figure 2; table 1). In total, 36.4% of individuals were estimated to undergo DVM at one time. *E. carlsbergi* exhibited the shortest migration distances with individuals present around 350 m depth at night and 400 m depth in the day (figure 2; table 1). Individuals were mainly confined to depths between 100 m and 600 m. This species also had the smallest proportion of migratory individuals at only 9.1%.

### (b) Total and migratory biomass of Southern Ocean myctophids

An all-species GAM was built to analyse the overall DVM pattern by myctophids in the Southern Ocean, which explained 15% of the model variance and indicated a clear DVM pattern (figure 3A). At night, myctophids were distributed from the ocean surface down to 400 m depth with the highest concentration around 350 m depth, while daytime depth ranged from 400 m down to 1000 m depth. The depth-integrated abundance was 0.448 individuals per m^2^ at night and 0.349 individuals per m^2^ for the day (table 1), which showed higher variability than at night (figure 3B). With an estimated area of 39.78 million km^2^ for the Southern Ocean (regions with ≥1000 m depth within the 48–65° S latitude boundary; figure 1), multiplying the area-specific total myctophid count generated an estimate of approximately 18 trillion fishes with a biomass of 48.3 Mt (95% CI [35.3, 67.4]), 0f which 29.3% of the population undertake DVM, equivalent to a biomass of 14.2 Mt. The standard length, wet weight and estimated proportion of each species within the population are summarized in table 2.

**Figure 3.**
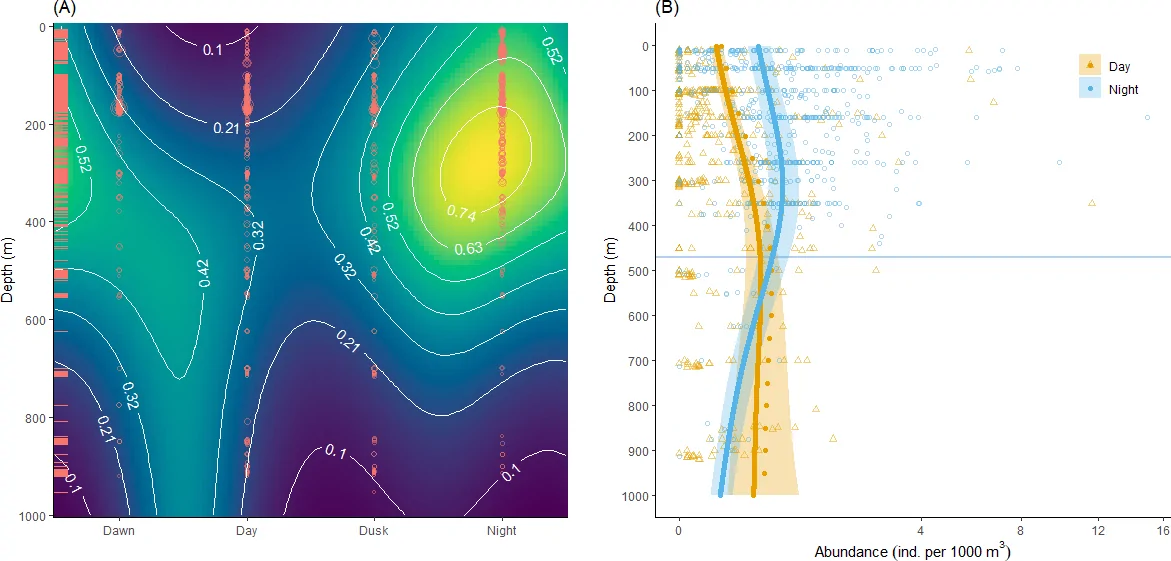
(A) Abundance (individuals per 1000 m^3^) of all eight myctophid fish species in the Southern Ocean, predicted from an all-species GAM across the studied depth range (*y*-axis) at different times of the day (*x*-axis). The rug on the left-hand side shows the density of data used to build the model. The mean abundances calculated from raw data are shown by circles over the heatmap with size reflecting abundance. (B) Predicted (line) and observed (points) abundance of all eight myctophid fish species by an all-species GAM along the ocean depth at daytime (solid orange) and night-time (blue). The dashed orange line shows an inflated daytime abundance, where the total depth-integrated abundance was equalized for day and night. The blue horizontal line represents the threshold depth used to identify individuals undergoing DVM. Shaded regions represent 95% confidence intervals. The *x*-axis is presented on a square root scale for clarity.

**Table 2.** Total myctophid abundance and biomass in the Southern Ocean (48–65°S) predicted from the all-species GAM. Standard length (SL) and mass (wet weight, WW) show the estimated SL and WW at the median sampled latitude and depth with a net effect of 0. Empirical abundance was calculated from the original sample data taking the mean at each latitude and then averaging across all latitudes. Proportion shows the proportion of each species within the total myctophid community of the study species. Abundance is the depth-integrated (1–1000 m) night-time abundance predicted from GAM, which was then multiplied by WW to derive biomass. Total abundance and total biomass show the estimated total in the Southern Ocean, with biomass estimated from the proportions of each species in the myctophid community and their WW. 95% CI are shown in the parentheses, calculated as the sum of lower CIs at each 1 m depth interval and the sum of upper CIs at each 1 m depth interval, respectively.

species	SL (mm)	WW (g)	empirical abundance (ind. per 1000 m^3^)	proportion of myctophid community	abundance (ind. m^−2^)	biomass (g m^−2^)	total abundance (trillion ind.)	total biomass (million tonnes)
*Electrona antarctica*	56.8	2.2	0.352	0.50	0.222 [0.162–0.310]	0.489 [0.357–0.681]	8.83 [6.46–12.32]	19.44 [14.21–27.1]
*Krefftichthys anderssoni*	47.1	1.1	0.148	0.21	0.093 [0.068–0.130]	0.102 [0.075–0.143]	3.70 [2.71–5.16]	4.07 [2.98–5.68]
*Gymnoscopelus braueri*	82.5	4.1	0.083	0.12	0.052 [0.038–0.073]	0.214 [0.156–0.298]	2.07 [1.51–2.89]	8.49 [6.21–11.84]
*Protomyctophum bolini*	47.5	1.4	0.041	0.06	0.026 [0.019–0.036]	0.036 [0.026–0.050]	1.03 [0.75–1.43]	1.44 [1.05–2]
*Gymnoscopelus nicholsi*	137.6	25.1	0.010	0.01	0.007 [0.005–0.009]	0.165 [0.121–0.230]	0.26 [0.19–0.37]	6.57 [4.8–9.16]
*Gymnoscopelus fraseri*	76.2	4.5	0.017	0.02	0.011 [0.008–0.015]	0.048 [0.035–0.067]	0.42 [0.31–0.59]	1.90 [1.39–2.65]
*Protomyctophum tenisoni*	57.4	2.0	0.030	0.04	0.019 [0.014–0.027]	0.038 [0.028–0.054]	0.76 [0.56–1.07]	1.53 [1.12–2.13]
*Electrona carlsbergi*	78.5	6.6	0.030	0.04	0.019 [0.014–0.026]	0.123 [0.090–0.172]	0.74 [0.54–1.03]	4.89 [3.58–6.82]
Total			0.711		0.448 [0.328–0.625]^a^	1.215 [0.888–1.694]	17.82 [13.03–24.85]^a^	48.34 [35.33–67.39]

a Predictions from an independent all-species GAM, rather than the sum of the eight species-level models.

## 4. Discussion

### (a) Diel vertical migration behaviour of key myctophid species

Our GAMs predicted a clear DVM pattern in all eight species with deeper vertical distribution during the day than at night. The deepest migrations were undertaken by *Electrona antarctica*, *Krefftichthys anderssoni* and *Gymnoscopelus braueri*, extending to below 500 m depth on average during the day. Species differed in the extent of their migrations. On average, *Gymnoscopelus braueri* travelled the furthest, migrating from below 700 m depth in the day to roughly 300 m depth at night. *Krefftichthys anderssoni* displayed a unique daytime distribution with high abundances found at both epipelagic layer and at >800 m depth. Moderate DVM of an average 180 m distance was shown by *Gymnoscopelus fraseri* and *Protomyctophum tenisoni*, while G*ymnoscopelus nicholsi* and *Protomyctophum bolini* displayed DVM of 100 m distance on average. The least extensive DVM was seen in *Electrona carlsbergi*, which showed an average population-DVM of less than 50 m distance. The estimated vertical distribution by our model is generally in agreement with past observations [2–4,21,23].

Myctophid species undergo DVM for predator avoidance and feeding [38], and their vertical distributions overlap with those of their prey items [9,39]. The high variability in DVM extent observed among species may be partly driven by feeding preferences. Species such as *E. antarctica* and *G. nicholsi* may come to surface waters to feed on Antarctic krill (*Euphausia superba*), their main prey that are largely confined to the upper 200 m of the water column [40]. Whereas species like *P. bolini* that share a vertical distribution range with its main prey, the copepod *Metridia gerlachei*, may only require a shallow DVM to feed [39]. The variation in depth occupation and DVM extent among myctophids has previously been proposed to enable niche separation in the open ocean habitat, where closely related myctophid species often overlap in latitudinal distribution [41] and zooplankton are utilized by multiple predator species [21,23,42]. Such interspecific differences in depth occupancy may also be driven by contrasting environmental preferences for dissolved oxygen levels, sea surface temperature and sea ice concentration [28,43]. An important caveat of our models is that they are based on summer-biased data, and therefore, our results may not capture seasonal variation in DVM that has been observed in some species [22,23]. In addition, our models do not account for ontogenetic variation in depth distribution [3,19,21], which was observed in five of the study species (results and discussions in electronic supplementary material, figures S4 and S5). Since many of the variables presumed to affect DVM tend to covary with one another, approaches such as mechanistic modelling may be valuable for identifying causal factors controlling DVM behaviour (e.g. [44,45]).

With the exception of *P. bolini*, night-time abundance was consistently higher than daytime abundance, indicating daytime net avoidance [2,3]. Net-based abundance estimates of myctophids are an order of magnitude lower than those estimated by active acoustic methods [1,46], so visual net avoidance is likely to be a contributor to the low daytime surface abundance shown by our model. Day–night discrepancies in abundance were most prominent in low-abundance species, making it difficult to discern whether these species are actually low in abundance or are more effective at net avoidance. Behaviours such as schooling may also affect catch efficiency. The volume filtered in sampling was not equal across the study, and shorter hauls may have an elevated risk of missing shoaling species such as *K. anderssoni* and *E. carlsbergi* [47]. Alternative sampling approaches, such as the use of eDNA-based methods, may provide further insight into the abundance of these species [48,49].

Importantly, trawl gears are known to vary in their sampling efficiency. Large nets towed at high speed can catch larger, fast-swimming fish but are prone to missing smaller individuals, which can only be retained by smaller, finer-meshed nets [24–26]. This was apparent in our analysis, where the smaller RMT8 net (rectangular midwater trawl with 8 m^2^ mouth-opening) was apparently less effective than the larger RMT25 net at catching most species, but was the only net that caught juvenile *E. antarctica* smaller than 20 mm standard length (electronic supplementary material, figure S9). The combination of nets with complementary characteristics in our study ensures fish at all life stages would be caught, allowing a more comprehensive analysis of their distribution and behaviour. While there are potential risks in undertaking quantitative analysis from nets that differ in catch efficiency [24], our analysis partially mitigated net biases by including net characteristics as a predictor variable in the models. However, since the use of different nets was not even across the Southern Ocean (figure 1), some sampling biases likely resulted from the spatial variability of net characteristics instead of myctophid distribution. Notably, Woods *et al.* [28] found minimal influence of net type and mesh size when making predictions of abundance using data sourced from Myctobase.

### (b) Variable diel vertical migration behaviour

Not all individuals within the myctophid community undergo DVM. A proportion of each species was found to remain towards the surface in the daytime, while another proportion remained in deeper waters at night. For most species, roughly a third of the individuals appear to undergo DVM at one time, defined as the estimated abundance of individuals that crossed the species-specific threshold depth. The species with the highest proportion of vertical migrants was *G. braueri*, where nearly 60% of the population migrated, while the species with the lowest proportion was *E. carlsbergi*, where less than 10% of the population migrated.

To calculate these proportions of individuals undergoing DVM in each species, we adjusted daytime abundance estimates to account for daytime net avoidance, assuming a uniform effect of net avoidance across depth. However, the net avoidance effect is likely to be stronger near the surface where light intensity is higher, so a uniform inflation may underestimate the daytime surface abundance and overestimate the proportion of migrating population. To assess the sensitivity of this assumption, a depth-dependent inflation based on light attenuation was applied, where daytime abundance was only inflated above certain depth thresholds to match the total night-time abundance (174 m, 477 m and 780 m chosen by decreasing light availability). Under this approach, daytime surface abundance exceeded night-time surface abundance for some species when the 174 m threshold was applied, and the estimated proportion of migrating population was reduced by 60% and 20% on average across the eight species for the 477 m and 780 m thresholds, respectively (electronic supplementary material, figure S10). But such depth-dependent inflation introduces additional assumptions regarding the relationship between light and net avoidance that cannot be evaluated with the available data. To our knowledge, there are currently no empirical studies that quantified the relationship between net avoidance and depth/light that can be used to calculate reliable daytime inflation, and it would be valuable to improve future calculations of myctophid DVM with such information. There is also the possibility that some species may have migrated deeper than the 1000 m threshold used in our analysis, leading to lower daytime abundance estimates. In general, deeper trawl samples below 600 m are scarce, even in the large dataset used in our study, which constrains our understanding of DVM in the deeper sectors of the mesopelagic zone.

The broad vertical distribution of myctophids during the night has been observed in both net trawl studies [3,21,28,50] and acoustics [43]. It is possible that some individuals are not surfacing and remain at depth. Acoustic studies have identified non-migratory individuals within the North Atlantic myctophid *Benthosema glaciale* [51,52]. This is compatible with the gut fullness analysis of *B. glaciale* that revealed they only feed once every 2 days, plausibly because species with low metabolic rates may not require daily feeding, enabling individuals to remain at depth and avoid the risk of surface predation [53]. Importantly, *E. antarctica* and *G. braueri* have also been found to have very low stomach fullness in some circumstances [39], which could be an indication of non-daily feeding. As these species showed the longest migration distances in our study, it may be too energetically costly for them to perform frequent DVMs. Another possibility for the broad distribution of individuals observed at night is that individuals are migrating multiple times throughout the night—termed ‘continuous foray’. This behaviour was apparent in *P. bolini*, which has been observed to have two peaks in stomach fullness during the hours of darkness, linked to multiple feeding events at night [39]. Continuous feeding may also contribute to the presence of daytime surface-caught adult individuals in some species. Daytime feeding has been identified in *G. nicholsi*, which has been found with fresh food in stomachs when caught during the day [39]. Individuals may also be compelled to surface in daytime to allow for visual foraging [52]. Such a reverse DVM pattern has been observed in *B. glaciale* [52] and may explain the daytime surface population of *K. anderssoni* observed in our result. With a global distribution except the Arctic, myctophid fishes display a range of DVM patterns across the world [38,54]. The variable DVM patterns shown in our result, where only a fraction of the population undergoes normal DVM, are concurrent with observations from other parts of the oceans [52,55,56]. In contrast, synchronous DVM, where the whole population migrates together, is often limited to less-productive regions of the sea, where the limited feeding opportunity forces the whole population to travel to the feeding area (e.g. [57]).

Variation in vertical migration strategies has potentially strong implications for the role of myctophids in carbon export. Current estimates of myctophid-driven carbon export have been made on the assumption of a single migration per night [14,15], so research may have underestimated myctophid contributions to the deep-ocean carbon sink, as has been the case for krill [58]. The reverse is also possible that myctophid carbon transport has been overestimated if DVM occurred less than once per night. Assumptions of synchronous migration and daily feeding of myctophids have also been widely applied in trophodynamic studies when estimating their consumption rate of prey items [6,59,60], which could potentially under- or overestimate their role in the food web, depending on their actual feeding strategies. Assumptions of single night-time DVM across species may also underestimate daytime prey availability to surface visual predators such as grey-headed albatrosses [61,62]. While individuals are likely to differ in their DVM strategies, we are unable to discern between them with our data, which only shows population-scale movement. The same issue also applies to bioacoustic measurements. This highlights the need for more research using approaches such as gut content analysis, for an improved understanding of the movement biology of individuals within myctophid populations to fully understand their role in Southern Ocean ecosystems [63].

### (c) Total biomass and transfer by diel vertical migration

The total abundance of the eight myctophid species in our study range of the Southern Ocean was estimated as 18 trillion fishes, equating to a total biomass of 48.3 million tonnes. The estimated Southern Ocean myctophid biomass in our study, therefore, is equivalent to over half of the global capture fishery production [64], highlighting the vast biomass of mesopelagic fish held in the study region, which only encompasses approximately 3% of the global ocean volume [65]. In total, *E. antarctica* accounted for 50% of this community, followed by 20 and 12% for *K. anderssoni* and *G. braueri,* respectively. Our estimate of myctophid biomass (1.21 g per 1000 m^3^) is lower than previous net-based estimates from the Scotia Sea (2.93 g per 1000 m^3^ [3]; 2.23 g per 1000 m^3^ [2]) and Macquarie Island (1.59 g per 1000 m^3^ [19]). The lower estimate in our study likely reflects our use of estimates of average biomass through the water column from the surface to 1000 m depth, rather than net catches that can show biases towards shallow waters (electronic supplementary material, figure S5). The lower estimate in our study may also be partially due to the use of only eight myctophid species, whereas values reported in the three studies cited above [2,3,19] included additional myctophid species in their biomass estimates (although our focal species collectively comprise >85% of the myctophid individuals reported in those studies). Notably, biomass estimates for individual species were similar to or higher in our study than in some other studies [3,10]. The high productivity of the Scotia Sea is also likely to have contributed to its particularly high abundance of myctophids [2,3]. Since the species analysed in our study have a circumpolar distribution [4,28], latitudinal differences in species composition could also play a role in the lower values we report relative to other studies. Myctophid diversity reduces towards the pole with many species in greatest abundance around the Polar Front [2,66]. Our lower biomass estimate reflects the reduced abundance of larger species in the high latitudes. This highlights the need for caution when scaling regional estimates to the whole of the Southern Ocean, and ideally, estimates would consider latitudinal differences in community structure. Although the dataset used in our study covered a wide latitudinal range, it still had significant gaps in its spatial coverage, namely from the Ross Sea sector. Future sampling efforts focused on less comprehensively studied regions would help to gain a better understanding of the spatial variability in both myctophid distribution and DVM behaviour.

Acoustic estimates of Southern Ocean myctophid biomass have ranged between 201 Mt and 398 Mt [67], which are considerably greater than all net-based estimates. While acoustic studies do not suffer from the problem of net avoidance [46], there are still considerable uncertainties in their biomass estimate due to the difficulties in distinguishing myctophids from other organisms in the acoustic backscatter and converting these backscatters attributed to myctophids into biomass [1,67,68]. Combining net-based biomass estimates with acoustic estimates helps to reduce these uncertainties, whereby the two approaches provide complementary lower and upper boundaries to myctophid biomass estimates, respectively.

About a third of the myctophid community was estimated to perform DVM daily, equating to 14.2 Mt of daily biomass movement by myctophid DVM. Applying this proportion to acoustic estimates of myctophid biomass suggests 59–117 Mt of daily biomass movement [67]. Such large daily transport of biomass could have a significant contribution to carbon export [1]. As the three most abundant myctophid species were also those that underwent the greatest migrations, the overall DVM distance was extensive—ranging from the epipelagic at night to below 500 m in the day. Remineralization of carbon has been shown to decline when released below 500 m depth [69,70], indicating that carbon transported by myctophids at depth will likely reach deep water for long-term sequestration. It should be noted, however, that the DVM extent predicted by our model is the average movement across all latitudes. Change in fish size along latitudes was observed in some species, which could affect the DVM pattern (results and discussions in electronic supplementary material, figures S4 and S5). Reduction in DVM at higher latitudes has been reported from both poles [36,71,72], so the efficiency of carbon transport may be reduced at higher latitudes if the myctophid community does not dive as deep.

Currently, it is estimated that fish respiration contributes to an equivalent of 27–143% of passive carbon flux (gravitational sinking of organic matter) to the Southern Ocean carbon pump [14,15], but this is likely to be an underestimate as it does not include carbon export through their egestion, or the decomposition of their carcasses upon mortality (deadfall). The vertical transport of biomass through myctophid DVM is also critical from a trophodynamics perspective, connecting energy pathways between surface and deep waters [9]. This is of particular interest, since energy transfer via myctophids is likely to increase in importance as the observed decline in krill threatens the integrity of the established diatom-krill-higher predator pathway in the Southern Ocean [9,73,74]. The DVM patterns resolved in our study provide a useful framework to better understand future changes to myctophid-based ecosystem processes in the Southern Ocean. Key knowledge gaps remain in understanding the variation in daily vertical movements among individuals of the most ecologically important species, which may impact both carbon export and ecosystem processes.

## Supplementary Material



## Data Availability

All data analysed in this study are publicly available. Myctobase [27] can be found on Zenodo [75]. GEBCO gridded database can be downloaded [34]. The codes used in this paper can be found at Zenodo [76]. Supplementary material is available online [77].

## Supplement

Electronic supplementary material is available online at https://doi.org/10.6084/m9.figshare.c.8288865.
